# Association between serum zinc level and lipid profiles in children with spinal muscular atrophy

**DOI:** 10.3389/fnut.2022.960006

**Published:** 2022-08-15

**Authors:** Qi Long, Yijie Feng, Fei Chen, Wenqiao Wang, Ming Ma, Shanshan Mao

**Affiliations:** ^1^Department of Clinical Nutrition, The Children's Hospital, Zhejiang University School of Medicine, National Clinical Research Center for Child Health, Hangzhou, China; ^2^Department of Neurology, The Children's Hospital, Zhejiang University School of Medicine, National Clinical Research Center for Child Health, Hangzhou, China

**Keywords:** spinal muscular atrophy, dyslipidaemia, serum zinc, children, high-density lipoprotein cholesterol, apolipoprotein A1

## Abstract

**Background and aims:**

Children with spinal muscular atrophy (SMA) have a high rate of dyslipidaemia, which is a risk factor of vital importance for cardiovascular diseases in adulthood. Studies have demonstrated that the serum zinc level is associated with lipid profiles in the general population as well as in individuals diagnosed with obesity or diabetes. The purpose of this study was to evaluate the relationship between serum zinc level and lipid profiles in children with SMA.

**Methods:**

This cross-sectional study was launched in a tertiary children's medical center in China and involved pediatric patients with SMA under the management of a multidisciplinary team of outpatient services from July 2019 to July 2021. Anthropometric information, general clinical data, serum zinc level, lipid profiles, and body composition data were collected. Multivariate analysis was used for a thorough inquiry on the association between the serum zinc level and lipid profiles.

**Results:**

Among the 112 patients with SMA [median (IQR) age 5.54 years (2.75–8.29), 58.04% female], who fulfilled the inclusion criteria of the study, dyslipidaemia was detected in 60 patients (53.57%). Based on multivariable linear regression, serum zinc level was positively associated with high-density lipoprotein cholesterol (HDL-C; β = 1.63, 95% CI = 0.44–3.22) and apolipoprotein A1 (APO A1; β = 2.94, 95% CI = 0.03–5.85) levels, independently of age, sex, type, activity, percentage of body fat, and body mass index. As the serum zinc level increased by 10 μmol/L, the risk of low APO A1 levels decreased by 35% (OR = 0.65, 95% CI = 0.44–0.97) according to multivariable logistic regression analyses.

**Conclusion:**

Serum zinc concentration was positively correlated with HDL-C and APO A1 levels among children with SMA. We suggest measures to correct the lower level of serum zinc to improve HDL-C and APO A1 levels.

## Introduction

Spinal muscular atrophy (SMA) is a rare genetic neuromuscular disease with an autosomal recessive inheritance pattern. The most common type is 5qSMA, which is caused by a homozygous mutation or deletion in the survival motor neuron 1 gene positioned on chromosome 5q11.2–q13.3 (1). The worldwide incidence of SMA is ~1/10,000 live births, and the carrier frequency is 1/42 in the Chinese population (2, 3). SMA manifests as progressive muscle weakness and atrophy resulting from motor neurons' pathological degeneration in the anterior horn of the spinal cord, which is often accompanied by organ damage in the respiratory, digestive, and skeletal systems (4).

In recent years, drug treatment for SMA has achieved unprecedented progress (5). Despite medication development, experts have unanimously pointed out that SMA standardized treatment and individualized management always require the cooperation of a patient-centered multidisciplinary team and that nutrition management is indispensable (6, 7). Over the last few years, research in SMA nutrition has focused mainly on evaluating the nutritional status, body composition, dietary intake, bone health, and individual energy demand (8, 9). Moreover, recent literature has described metabolic abnormalities in SMA, of which dysregulation of the lipid profiles is the first and most frequently studied issue (10–12). Studies on lipid metabolism in patients with SMA have confirmed abnormal levels of fatty acid oxidative metabolites and increased susceptibility to dyslipidaemia (11, 13).

Prevention and treatment of dyslipidaemia is a challenging clinical issue. The therapeutic management of childhood dyslipidaemia mainly consists of lifestyle adjustment, emphasizing suitable dietary patterns, and increased physical activity for early prevention of atherosclerosis and coronary artery diseases (14–16). However, in children with SMA, improvement through exercise is more difficult because of motor dysfunction.

Zinc is considered one of the essential micronutrients for the human body and plays a significant role in the metabolism of lipids, proteins, and carbohydrates (17). Studies have shown that individuals with an inordinate amount of body fat have lower serum zinc levels (18). Research shows that, in comparison with the general population, patients with SMA often have a decreased fat-free mass and an increased fat mass (19). However, little research has been conducted on serum zinc levels in patients with SMA. It has been implied that zinc insufficiency may disrupt the energy generation process, and thus adipose tissue is shaped (17). Under conditions of low blood zinc level, lipid management is impaired, causing a rise in triglycerides (TG), total cholesterol (TC), and low-density lipoprotein cholesterol (LDL-C), as well as a decrease in high-density lipoprotein cholesterol (HDL-C) levels in adults, which in turn leads to obesity, metabolic syndrome, and diabetes (20–23). Zinc supplementation can improve blood lipid levels, thus reducing morbidity and mortality associated with cardiovascular diseases (24).

This study was designed to explore the potential relationship between serum zinc level and lipid profiles in children with SMA and provides new treatment strategies for reducing the incidence of dyslipidaemia.

## Materials and methods

### Subjects and study design

**Figure 1:**
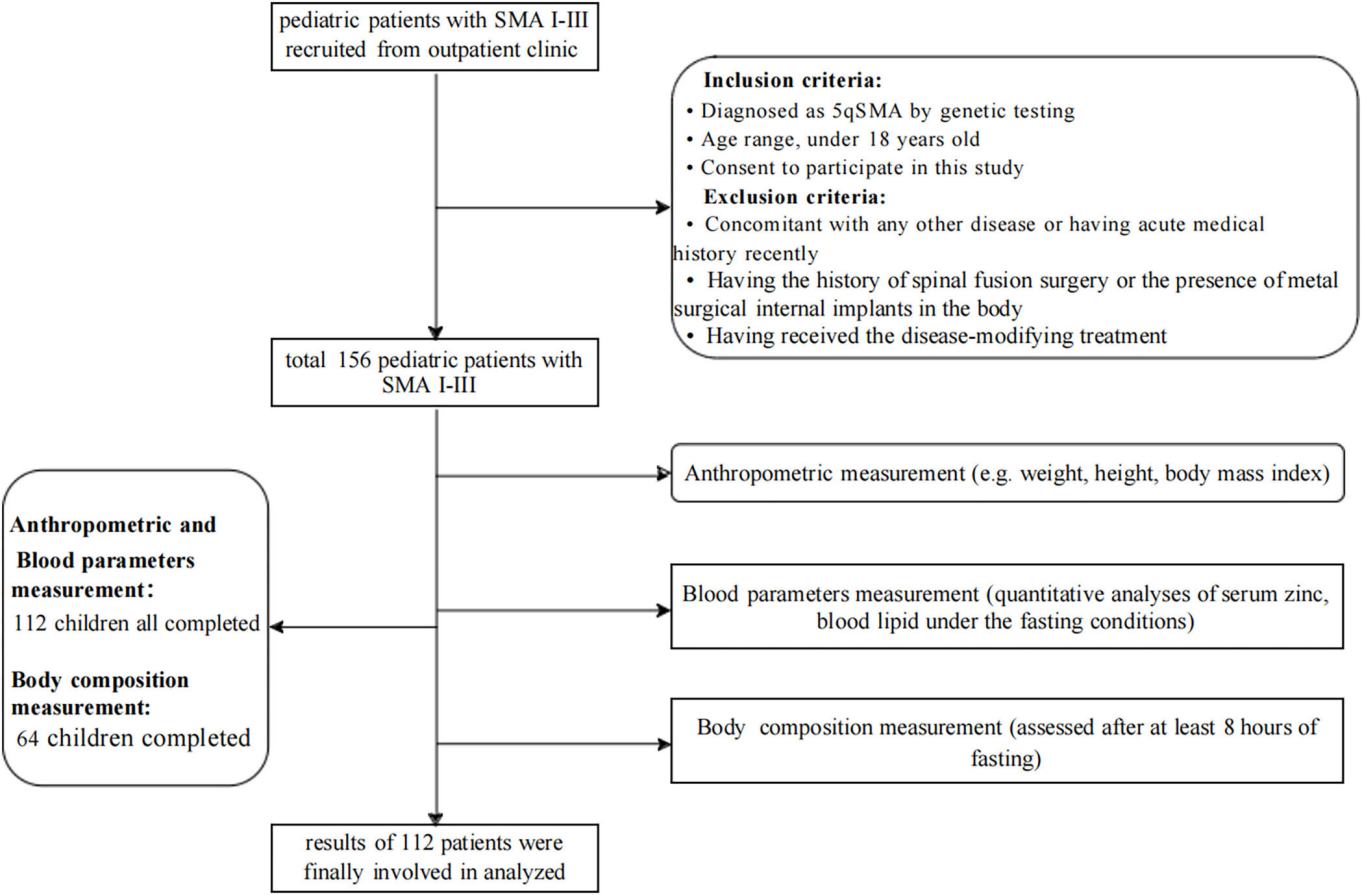
Flow chart showing patient selection.

This cross-sectional study was prospectively carried out in an outpatient department of our hospital from July 2019 to July 2021. The inclusion criteria were as follows: (1) diagnosis of 5qSMA by genetic testing; (2) age < 18 years; (3) provision of consent to take part in this study. Those who met the following criteria were excluded: (1) concomitant presence of any other disease or a recent acute medical history; (2) a history of spinal fusion surgery or the presence of metal surgical implants in the body; and (3) having received disease-modifying treatment. Ethical approval was obtained from the Ethics Committee of the Children's Hospital of the Zhejiang University School of Medicine (2019-IRB-171). Written informed consent was acquired from the participants and their guardians. The selection procedure is illustrated in Figure 1; a total of 112 patients were finally included.

### Measurements of exposures (independent variables)

Quantitative analyses of serum zinc levels were performed utilizing a multichannel atomic absorption spectrophotometer-MB5 (Beijing Persee General Instrument Company Limited, Beijing, China).

### Study outcomes (dependent variables)

Blood samples for lipid tests were collected under fasting conditions. Serum TG, TC, APO A1, APO B, HDL-C, and LDL-C levels were measured using an automatic biochemical analyser (Beckman Coulter Inc., USA). The cut-off points for lipids recommended by the National Cholesterol Education Program were as follows: LDL-C ≥130 mg/dl, TG ≥100 mg/dl (0–9 years), TG ≥130 mg/dl (10–19 years), TC ≥200 mg/dl, HDL-C <40 mg/dl, APO B ≥110 mg/dl, and APO A1 <115 mg/dl (25).

### Covariates

The covariates in this study included age, sex, body mass index (BMI), disease classification, activity, and percentage of body fat. Anthropometric data were collected by professionally trained clinical research coordinators. Body weight was measured using a lever scale (electronic scale for babies) with an accuracy of 50 g. Height was measured using a baby height-measuring board for children under 2 years old and a height-measuring instrument with an accuracy of 0.1 cm for patients over 2 years old. When the patient could not stand, the height was replaced by the arm span. The BMI was computed as the ratio of weight to height squared (kg/m^2^).

Disease classification was performed according to the maximum athletic ability achieved. The degree of activity was based on currently achievable athletic ability. Fat mass was obtained by an InBody S10 body composition analyser (Biospace, Seoul, Korea). This assessment was carried out after at least 8 h of fasting with the patients in a sitting posture with the legs set apart and arms not touching the torso. The fat mass divided by the body weight was used as the percentage of body fat.

### Statistical analysis

Quantitative variables are described as median with range (quartile) or mean ± standard deviation, and frequencies with percentages are used to describe binary variables. The serum zinc level cut-off was taken as 75 μmol/L. We divided the patients into two groups based on their serum zinc median level. To compare the differences between the two groups, we used chi-square tests or Fisher's exact test for categorical variables and Student's *t*-test or the Mann–Whitney *U*-test for quantitative variables. Moreover, logistic and linear regression models were applied to analyse the association between the serum zinc level and lipid profiles adjusted for major covariates, including age, sex, BMI, disease classification, activity, and percentage of body fat (univariate and multivariate analyses). Multiple imputations with chained equations accounted for the missing data on the percentage of body fat. Statistical software packages R (http://www.R-project.org, The R Foundation) and Empowerstats (http://www.empowerstats.com, X&Y Solutions, Inc., Boston, MA, USA) were utilized to perform the analyses mentioned above.

## Results

### Clinical characteristics of patients

A total of 112 participants (65 women and 47 men) were enrolled: 16 with type I, 57 with type II, and 39 with type III SMA. The baseline characteristics of the study participants, divided by the median serum zinc level, are shown in Table 1. No differences were observed in sex, activity, BMI, or percentage of body fat (all *P* < 0.05), whereas differences in the age and types of SMA between the two groups (all *P* > 0.05) were found to be significant. Over half (53.57%) of the patients with SMA tested positive for at least one out of the six indicators for laboratory-defined dyslipidaemia (Table 1). The prevalence of abnormal TC, LDL-C, HDL-C, TG, APO A1, and APO B levels ranged from 4.46 to 25.90%. We found that TG, HDL-C, and APO A1 levels in the two groups were significantly different (i.e., serum zinc levels increased with a decrease in TG and an increase in HDL-C and APO A1 levels).

**Table 1:** Baseline characteristics of the study participants by the median level of serum zinc.

	**Serum zinc (**μ**mol/L)**			
**Variables**	**Total** **(*n* = 112)**	< The median level of serum zinc* **(*n* = 56)**	≥The median level of serum zinc* **(*n* = 56)**	***P*-value**
Age (years)	5.54 (2.75–8.29)	3.12 (1.63–5.71)	7.33 (5.35–11.42)	<0.001
**Sex**
Female	65 (58.04%)	31 (55.36%)	34 (60.71%)	0.566
Male	47 (41.96%)	25 (44.64%)	22 (39.29%)	
**SMA type**
I	16 (14.29%)	11 (19.64%)	5 (8.93%)	0.024
II	57 (50.89%)	32 (57.14%)	25 (44.64%)	
III	39 (34.82%)	13 (23.21%)	26 (46.43%)	
**Activity**
Non-sitters	17 (15.18%)	11 (19.64%)	6 (10.71%)	0.103
Sitters	57 (50.89%)	31 (55.36%)	26 (46.43%)	
Walks
Body mass index, (kg/m^2^)	15.49 ± 3.48	15.59 ± 2.86	15.39 ± 4.03	0.763
Percentage of body fat, (%)	36.12 ± 12.17	37.13 ± 11.11	35.43 ± 12.94	0.587
TG, (mg/dl)	90.14 ± 46.44	98.98 ± 49.10	81.31 ± 42.21	0.043
**TG** **≥100 mg/**dl
No	83 (74.11%)	37 (66.07%)	46 (82.14%)	0.052
Yes	29 (25.89%)	19 (33.93%)	10 (17.86%)	
TC, mg/dl	167.77 ± 55.75	166.74 ± 69.50	168.80 ± 37.96	0.846
**TC** **≥200 mg/**dl
No	98 (87.50%)	50 (89.29%)	48 (85.71%)	0.568
Yes	14 (12.50%)	6 (10.71%)	8 (14.29%)	
HDL-C, mg/dl	47.38 ± 10.87	45.17 ± 10.64	49.56 ± 10.74	0.033
**HDL-C** ** <40 mg/**dl
No	88 (78.57%)	41 (73.21%)	47 (83.93%)	0.167
Yes	24 (21.43%)	15 (26.79%)	9 (16.07%)	
LDL-C, mg/dl	101.32 ± 28.25	101.78 ± 33.09	100.88 ± 22.82	0.867
**LDL-C** **≥130 mg/**dl
No	99 (88.39%)	48 (85.71%)	51 (91.07%)	0.376
Yes	13 (11.61%)	8 (14.29%)	5 (8.93%)	
APO A1, mg/dl	124.67 ± 19.72	119.73 ± 21.14	129.52 ± 17.06	0.008
**APO A1** ** <115 mg/**dl
No	83 (74.11%)	35 (62.50%)	48 (85.71%)	0.005
Yes	29 (25.89%)	21 (37.50%)	8 (14.29%)	
APO B, mg/dl	74.12 ± 24.43	72.84 ± 24.78	75.38 ± 24.23	0.586
**APO B** **≥110 mg/**dl
No	107 (95.54%)	54 (96.43%)	53 (94.64%)	0.647
Yes	5 (4.46%)	2 (3.57%)	3 (5.36%)	
**Number of abnormal lipids**
0	52 (46.43%)	19 (33.93%)	33 (58.93%)	0.050
1	27 (24.11%)	18 (32.14%)	9 (16.07%)	
2	18 (16.07%)	10 (17.86%)	8 (14.29%)	
3	11 (9.82%)	5 (8.93%)	6 (10.71%)	
4	3 (2.68%)	3 (5.36%)	0 (0.00%)	
≥5	1 (0.89%)	1 (1.79%)	0 (0.00%)	

SMA, spinal muscular atrophy; TG, triglycerides; TC, cholesterol; HDL-C, high-density lipoprotein cholesterol; LDL-C, low-density lipoprotein cholesterol; APO A1, apoprotein A1; APO B, apoprotein B.

Values are n/N (%), median (IQR), and mean ± standard deviation. Among the 112 participants, the amount of missing data was 48 for the percentage of body fat.

*The median level of serum zinc: 75 μmol/L.

### Univariate analysis related to lipid profiles

To identify factors related to lipid profiles, we set each of the candidates as independent variables while using lipid profiles as the dependent variable (Table 2). Univariate analysis showed that age (β = −2.79, 95% CI = −4.55 to −1.03), activity (sitters vs. non-sitters: β = −25.68, 95% CI = −50.59 to −0.77), SMA type (type 2 vs. type 1: β: −26.90, 95% CI = −52.39 to −1.41), and zinc level (β = −0.63, 95% CI = −1.17 to −0.09) were associated with TG level. Zinc level was associated with HDL-C (β = 0.16, 95% CI = 0.04–0.29) and APO A1 (β = 0.28, 95% CI = 0.05–0.51) levels.

**Table 2:** Univariate analysis related to lipid profiles.

	**Statistics**	**TG**	**TC**	**HDL-C**	**LDL-C**	**APO A1**	**APO B**
**Sex** (*n*, %)
Female	65 (58.04%)	Reference					
Male	47 (41.96%)	−2.33 (−19.83, 15.17) 0.7944	20.79 (0.13, 41.44) 0.0510	−0.88 (−4.99, 3.23) 0.6753	5.20 (−5.44, 15.84) 0.3400	−0.82 (−8.28, 6.63) 0.8289	7.44 (−1.70, 16.57) 0.1135
Age	6.20 ± 4.72	−2.79 (−4.55, −1.03) 0.0024	−0.52 (−2.72, 1.69) 0.6476	0.33 (−0.09, 0.76) 0.1293	−1.14 (−2.24, −0.04) 0.0443	0.47 (−0.31, 1.25) 0.2378	−0.94 (−1.89, 0.01) 0.0546
**SMA type**
1	16 (14.29%)	0	0	0	0	0	0
2	57 (50.89%)	−26.90 (−52.39, −1.41) 0.0410	3.89 (−27.28, 35.06) 0.8071	3.63 (−2.43, 9.68) 0.2427	−2.95 (−18.68, 12.79) 0.7142	−0.36 (−11.40, 10.69) 0.9496	0.17 (−13.34, 13.68) 0.9804
3	39 (34.82%)	−22.25 (−49.00, 4.49) 0.1058	6.97 (−25.74, 39.68) 0.6771	2.50 (−3.84, 8.84) 0.4415	−8.73 (−25.22, 7.75) 0.3013	1.70 (−9.87, 13.27) 0.7741	−8.27 (−22.42, 5.88) 0.2544
**Activity**
Non-sitters	17 (15.18%)	0	0	0	0	0	0
Sitters	57 (50.89%)	−25.68 (−50.59, −0.77) 0.0458	2.27 (−28.14, 32.67) 0.8841	3.31 (−2.61, 9.23) 0.2756	−3.93 (−19.36, 11.50) 0.6186	−0.05 (−10.85, 10.74) 0.9926	−1.25 (−14.53, 12.03) 0.8542
Walkers	38 (33.93%)	−17.92 (−44.22, 8.39) 0.1847	9.30 (−22.81, 41.40) 0.5715	2.14 (−4.09, 8.38) 0.5021	−6.39 (−22.66, 9.87) 0.4425	1.51 (−9.87, 12.88) 0.7955	−7.10 (−21.09, 6.90) 0.3224
BMI	15.49 ± 3.48	1.81 (−0.67, 4.28) 0.1549	−1.63 (−4.61, 1.35) 0.2850	−0.59 (−1.17, −0.02) 0.0466	−0.53 (−2.05, 0.99) 0.4955	−0.95 (−1.99, 0.10) 0.0797	0.12 (−1.19, 1.44) 0.8556
(kg/m^2^)						
Percentage of body	36.12 ± 12.17	0.32 (−0.61, 1.25) 0.5011	−1.19 (−2.51, 0.13) 0.0815	−0.13 (−0.37, 0.10) 0.2772	−0.17 (−0.72, 0.37) 0.5353	−0.26 (−0.64, 0.13) 0.1940	0.20 (−0.32, 0.72) 0.4531
fat (%)					
Zinc (μmol/L)	77.56 ± 15.72	−0.63 (−1.17, −0.09) 0.0247	−0.21 (−0.87, 0.45) 0.5303	0.16 (0.04, 0.29) 0.0124	−0.12 (−0.45, 0.22) 0.4926	0.28 (0.05, 0.51) 0.0196	0.05 (−0.24, 0.34) 0.7381

TG, triglycerides; TC, cholesterol; HDL-C, high-density lipoprotein cholesterol; LDL-C, low-density lipoprotein cholesterol; APO A1, apoprotein A1; APO B, apoprotein B; SMA, spinal muscular atrophy; BMI, body mass index.

Data are represented as β (95% CI) and P-value.

### Independent relationship between serum zinc level and lipid profiles by multivariable linear and logistic regressions

We employed multivariable logistic regression analysis to demonstrate the association between serum zinc levels and dyslipidaemia (Table 3). The crude model analysis indicated that an increase of 10 μmol/L in serum zinc concentration was accompanied by a 36% decrease in the risk of an APO A1 level <115 mg/dl (OR = 0.64, 95% CI = 0.46–0.89). Further adjustment for age, sex, SMA type, activity, BMI, and percentage of body fat had little impact on the odds ratios of an APO A1 level <115 mg/dl (OR = 0.65, 95% CI = 0.44–0.95). In the crude model, elevated serum zinc levels reduced the risk of TG, HDL-C, LDL-C, and APO B dyslipidaemia, whereas, in models 1 and 2, the association was not significant (crude model; Models 1 and 2 in Table 3).

**Table 3:** Multivariable logistic regression between serum zinc and dyslipidaemia.

**Outcome**	**Crude model**	**Model 1**	**Model 2**
TG ≥100 mg/dl	0.71 (0.53, 0.97) 0.0298	0.88 (0.61, 1.25) 0.4681	0.89 (0.60, 1.33) 0.5745
TC ≥200 mg/dl	1.01 (0.71, 1.45) 0.9424	1.08 (0.71, 1.65) 0.7115	1.08 (0.70, 1.68) 0.7197
HDL-C <40 mg/dl	0.72 (0.52, 0.99) 0.0464	0.74 (0.50, 1.08) 0.1226	0.74 (0.49, 1.12) 0.1492
LDL-C ≥130 mg/dl	0.61 (0.39, 0.96) 0.0312	0.79 (0.46, 1.33) 0.3662	0.76 (0.44, 1.31) 0.3193
APO A1 <115 mg/dl	0.64 (0.46, 0.89) 0.0074	0.65 (0.44, 0.95) 0.0267	0.65 (0.44, 0.97) 0.0360
APO B ≥110 mg/dl	0.65 (0.50, 0.86) 0.0021	1.48 (0.75, 2.92) 0.2546	1.76 (0.80, 3.87) 0.1571
Dyslipidaemia	1.04 (0.59, 1.83) 0.8895	0.76 (0.55, 1.03) 0.0802	0.78 (0.56, 1.08) 0.1312

TG, triglycerides; TC, cholesterol; HDL-C, high-density lipoprotein cholesterol; LDL-C, low-density lipoprotein cholesterol; APO A1, apoprotein A1; APO B, apoprotein B; CI, confidence interval.

Multivariable logistic regression analysis of zinc and lipid levels. Data are presented as the OR (95% CI) P-value.

Outcome variables: TG ≥100 mg/dl, TC ≥200 mg/dl, HDL-C <40 mg/dl, LDL-C ≥130 mg/dl, APO A1 <115 mg/dl, APO B ≥110 mg/dl, and dyslipidaemia. Exposure variable: serum zinc level for each 10 μmol/L increase.

Crude model: no adjustment. Model 1: adjusted for age and sex. Model 2: adjusted for age, sex, SMA type, activity, BMI, and percentage of body fat.

We also used multivariable linear regression analysis to determine the association between the levels of serum zinc and lipid profiles (Table 4). In the crude model, serum zinc levels positively correlated with HDL-C (β = 1.63, 95% CI = 0.37–2.88) and APO A1 (β = 2.76, 95% CI = 0.48–5.05). After full adjustment, an increase of 10 μmol/L in serum zinc level led to a 1.63 mg/dl and 2.94 mg/L elevation in serum HDL-C (β = 1.63, 95% CI = 0.44–3.22) and APO A1 (β = 2.94, 95% CI = 0.03–5.85) levels, respectively (Table 4). In the crude model, serum zinc and TG levels were negatively correlated, whereas, in Models 1 and 2, the association was not significant. No linear association was observed between serum zinc and LDL-C, TC, or APO B levels (Table 4).

**Table 4:** Multivariable linear regression about serum zinc and lipid profiles.

**Outcome**	**Crude model**	**Model 1**	**Model 2**
TC, mg/dl	−2.12 (−8.74, 4.49) 0.5303	−2.29 (−10.14, 5.57) 0.5698	−2.91 (−11.15, 5.32) 0.4879
TG, mg/dl	−6.27 (−11.66, −0.87) 0.0247	−2.29 (−8.67, 4.08) 0.4824	−1.80 (−8.49, 4.90) 0.5986
HDL-C, mg/dl	1.63 (0.37, 2.88) 0.0124	1.57 (0.05, 3.08) 0.0457	1.63 (0.04, 3.22) 0.0449
LDL-C, mg/dl	−1.18 (−4.53, 2.18) 0.4926	0.92 (−3.06, 4.89) 0.6521	1.44 (−2.75, 5.63) 0.4999
APO A1, mg/dl	2.76 (0.48, 5.05) 0.0196	2.87 (0.10, 5.63) 0.0447	2.94 (0.03, 5.85) 0.0480
APO B, mg/dl	0.50 (−2.41, 3.40) 0.7381	2.80 (−0.58, 6.18) 0.1069	3.49 (−0.06, 7.03) 0.0541

TG, triglyceride; TC, cholesterol; HDL-C, high-density lipoprotein cholesterol; LDL-C, low-density lipoprotein cholesterol; APO A1, apoprotein A1; APO B, apoprotein B; CI, confidence interval.

Multivariable linear regression analysis of zinc and lipid levels. Data are presented as the β coefficient (95% CI) P-value.

Outcome variables: TG, TC, HDL-C, LDL-C, APO A1, and APO B.

Exposure variable: serum zinc level for each 10 μmol/L increase.

Crude model: no adjustment. Model 1: adjusted for age and sex. Model 2: adjusted for age, sex, SMA type, activity, BMI, and percentage of body fat.

## Discussion

This study, to the best of our knowledge, is the first study in this field to explore the association between serum lipids and serum zinc levels in children with SMA by adjusting for the effects of as many covariates as possible. Through the study, it was found that in our children patients with SMA, their serum lipid indicators level of them has different degrees of abnormality. Through the further detailed statistical analysis, we found that low serum zinc levels were significantly correlated with low levels of HDL-C and APO A1, which could provide a practical basis for further exploration of the influencing factors of lipid metabolism of SMA and management strategies for related complications.

Emerging studies have identified metabolic dysfunctions in patients with SMA, including lipid metabolic abnormalities, impaired glucose tolerance, and muscle mitochondrial defects (11, 12, 26). Studies have mostly focused on metabolic disorders involving fatty acid oxidation; however, some isolated studies have improved our understanding of fat metabolism. In a cohort study in Canada by Deguise et al. (13), the current prevalence of dyslipidaemia in 72 patients with SMA (16 with type 1, 52 with type 2, and 6 with type 3) was estimated to be 37.5%. Djordjevic et al. (26) determined the serum lipid profiles of 22 children with SMA type 2 and 15 with SMA type 3 and found 11 children (29.7%) with at least one indicator of abnormal lipid levels. Our study showed a higher incidence of dyslipidaemia (53.6%) than the previous two. This difference may be due to our larger sample size and the inclusion of APO A1 abnormalities in dyslipidaemia; 13% of the patients had over three laboratory-defined indicators of dyslipidaemia, similar to the prevalence reported in the cohort from Canada (14%) (13). In view of the large sample size and consistent results of the Canadian study with ours, we estimate that dyslipidaemia is an important yet undervalued feature of SMA. Dyslipidaemia can further complicate clinical conditions, leading to non-alcoholic fatty liver disease, cirrhosis, and cardiovascular and cerebrovascular diseases in adolescence and adulthood (14). Routine screening and management of SMA in patients with dyslipidaemia are necessary to reduce potential complications.

Zinc, an essential trace element and micronutrient, plays a pivotal role in human growth and development and participates in various physiological processes (17). The total prevalence of zinc deficiency in the general pediatric population is ~20%. There are still very few studies that have determined the dietary intake of zinc and serum zinc levels in patients with SMA. Our previous study confirmed that the frequency of insufficient zinc intake was 83.3% (27). Additionally, studies have consistently reported an increased fat mass and decreased fat-free mass in patients with SMA compared with healthy control subjects (28–30). Excess body fat conduces to increased cortisol synthesis and inflammation, which in turn induces Zip14 and metallothionein expression, causing a decrement in plasma zinc levels (21). Indeed, many studies have confirmed the lower levels of serum zinc in individuals who are overweight and who have obesity and diabetes (20, 21, 31).

Recently, researchers have also extended the correlation between zinc and blood lipid metabolism in the general population, adiposity, and patients with diabetes. However, these results remain controversial. No association (neither linear nor non-linear) was found between serum zinc levels and the risk of dyslipidaemia in participants in a National Health and Nutrition Examination Survey (32). A study by Rios-Lugo et al. (20) did not show any association between zinc concentration and cholesterol or TG levels in patients who were overweight and obese. In contrast, some studies demonstrated that serum zinc level was inversely associated with blood lipid levels (LDL, TG, and TC) and positively associated with HDL-C level in patients with diabetes type 1 or 2 (22, 23). In this study of 112 individuals with SMA, the association between zinc and APO A1/HDL-C levels was linear; that is, as the serum zinc level increases by 10 μmol/L, the HDL-C, and APO A1 levels will correspondingly increase by 1.63 and 2.95 mg/dl, respectively. It is also shown in this study that every 10 μmol/L increase in serum zinc level is associated with a 35% decrease in the incidence of APO A1 dyslipidaemia. However, in this model, the association between zinc levels and those of TC, LDL-C, TG, or APO B was not significant.

HDL-C and its main protein component, APO A1, play important roles in cholesterol homeostasis. The general functions of HDL-C and APO A1 are plasma-cholesterol transport, anti-inflammatory properties, and protection from oxidative damage. Low HDL-C and APO A1 levels are associated with the development of atherosclerosis (33). Patients with type III SMA who have entered adulthood may also experience coronary heart disease symptoms, such as angina pectoris, palpitations, syncope, and exertional dyspnoea (34). With the advent of targeted drugs for SMA, the lifespan of patients with SMA will be prolonged, and cardiovascular disease may probably become more prominent. Improving HDL-C level may contribute to reducing the incidence of coronary heart diseases in adults with SMA.

The potential mechanism through which zinc affects the lipid profile is still unclear. However, it has been proved that the biological mechanism of zinc involves lipid metabolism, suppression of reactive oxygen species production, and reduction in oxidative stress (17, 35). Therefore, appropriate zinc concentration may be essential for maintaining normal lipid levels. A meta-analysis has initially revealed that supplementation of zinc can increase HDL-C and reduce TC, LDL-C, and TG levels (24). Another review pointed out that zinc is an indispensable factor in regulating zinc alpha 2-glycoprotein (ZAG) homeostasis, which is considered an adipokine with anti-inflammatory and lipid-mobilizing activity (17, 18). Appropriate blood zinc concentrations are also relevant in maintaining appropriate ZAG activity as zinc facilitates the binding of adipokines to substrates, promoting lipolysis and lipid utilization (36). Tisdale et al. (37) demonstrated that ZAG could induce lipolysis both *in vivo* and *in vitro* by regulating triacylglycerol hydrolysis and free fatty acid release through signal transduction modulation in adipose tissue. A high level of ZAG can also increase HDL-C level *in vivo*, thereby participating in blood lipid metabolism (38). Combined with the above previous studies, the conclusion of our study preliminarily suggests that an appropriate increase in blood zinc levels in patients with SMA may help to regulate levels of blood lipids, providing evidence for the daily management of metabolism in children with SMA and for the research on the regulation mechanism of lipid metabolism in the future.

Our study had several limitations. First, as it was a cross-sectional observational survey, the cause– or time–effect relationships between serum zinc and lipid levels could not be clarified. Moreover, 42% of the patients did not complete BIA, and the accuracy of body composition analysis using BIA was not determined. However, we used multiple imputations with chained equations to account for the missing data on the percentage of body fat. We have adjusted for probability variables that may influence the result concerning the association between serum zinc and lipid levels. The results of the multivariable linear and logistic regressions were consistent, which further confirmed the relationship between zinc, HDL-C, and APO A1.

## Conclusion

Our findings show that among patients with SMA, low serum zinc level is frequent and associated with low levels of HDL-C and APO A1. Therefore, restored serum zinc levels may reduce the incidence of low HDL-C and APO A1 levels. Further studies are warranted to explore strategies to help reduce potential related complications of SMA.

## Data availability statement

The raw data supporting the conclusions of this article will be made available by the authors, without undue reservation.

## Ethics statement

The studies involving human participants were reviewed and approved by the Ethics Committee of the Children's Hospital of the Zhejiang University School of Medicine (2019-IRB-171). Written informed consent to participate in this study was provided by the participants' legal guardian/next of kin.

## Author contributions

SM and MM contributed to the conception and design of the research. QL contributed to the statistical analyses and manuscript writing. YF contributed to data collection and manuscript revision. FC and WW contributed to the data collection. All authors read and critically revised the manuscript and approved the final submission.

## Funding

This study was supported by the Key R&D Program of Zhejiang (2022C03167), the Zhejiang Province Public Welfare Technology Application Research Project (LGC21H090001), and the Key Technologies Research and Development Program of Zhejiang Province (2021C03099).

## Conflict of interest

The authors declare that the research was conducted in the absence of any commercial or financial relationships that could be construed as a potential conflict of interest.

## Publisher's note

All claims expressed in this article are solely those of the authors and do not necessarily represent those of their affiliated organizations, or those of the publisher, the editors and the reviewers. Any product that may be evaluated in this article, or claim that may be made by its manufacturer, is not guaranteed or endorsed by the publisher.
